# Supplementary material for: Content-rich biological network constructed by mining PubMed abstracts
Source: BMC Bioinformatics. 2004 Oct 8;5:147. doi: 10.1186/1471-2105-5-147 (PMC528731; doi:10.1186/1471-2105-5-147)
Supplement: Additional File 2 — The original results of the above study (non-essential files are deleted to keep the file size under the limit set by BMC bioinformatics). [file 1471-2105-5-147-S2.bz2 › chilibotAdditionalFile2/dip05/535ID10734072E10879/html/AR_CDK7.html]

 


 **AR** and **CDK7** 
  
Found 3 abstracts in PubMed, retrieved 3.  
 

 What does Google say? 
 PDF only 
| .edu only 

---

**Interactive relationship** (e.g. stimulation, inhibition, etc)

**Neutral relationship**- Detailed interaction studies further revealed that the  **AR**  NH 2 terminal domain interacting with CAK  [ **CDK7** ]  was essential for the CAK  [ **CDK7** ]  induced  **AR**  transactivation.  Ref: 10734072 J Biol Chem, 2000

**Non-interactive relationship** (e.g. studied together, co-existance, homology, etc.)

- Identification of cdk activating kinase  [ **CDK7** ]  CAK  [ **CDK7** ]  as an androgen receptor  [ **AR** ]  NH 2 terminal associated coactivator.  Ref: 10734072 J Biol Chem, 2000
- overexpression of Cdk activating kinase  [ **CDK7** ] , the kinase moiety of TFIIH, enhanced  **AR**  mediated transcription in prostate cancer cells.  Ref: 11266437 J Biol Chem, 2001
- Co transfection of cdk activating kinase  [ **CDK7** ]  CAK  [ **CDK7** ] , the kinase moiety of TFIIH, enhanced  **AR**  mediated transcription in a ligand dependent manner in human prostate cancer PC 3 and LNCaP cells, and in a ligand independent manner in human prostate cancer DU145 cells.  Ref: 10734072 J Biol Chem, 2000
